# IL-13 priming in precursors drives beige adipogenesis and enhances metabolic homeostasis

**DOI:** 10.1172/JCI191361

**Published:** 2025-06-02

**Authors:** Margo P. Emont, Jun Wu

**Affiliations:** 1 Department of Medicine, Section of Endocrinology, Diabetes and Metabolism, University of Chicago, Chicago, Illinois, USA.; 2 Life Sciences Institute and; 3 Department of Molecular and Integrative Physiology, University of Michigan, Ann Arbor, Michigan, USA.

## Abstract

Accumulating evidence from rodent and human studies indicates that the activity of thermogenic adipocytes positively correlates with optimal metabolic function. In this issue of the *JCI*, Yesian et al. uncover a paracrine signaling pathway from type 2 innate lymphoid cells to preadipocytes via IL-13 that increases beige adipogenesis through a PPARγ-dependent pathway. Mice with deletion of *Il13ra1* demonstrated glucose dysregulation, and variants near the human *IL13RA1* locus were associated with body weight and diabetic status. It is tempting to speculate that targeting IL-13 holds therapeutic potential for improving metabolic fitness in humans.

## Harnessing the therapeutic potential of beige fat

Since the discovery of activatable thermogenic fat in adult humans, effort has been made to better understand these unique adipocytes (1). Of particular interest is the question of whether thermogenic fat can be activated to treat metabolic disease (2). Review of patient records has indicated that increased thermogenic fat is associated with metabolic health (3), and studies have shown that β3 adrenergic receptor agonism can activate thermogenic fat, improving glucose tolerance (4, 5). Nonetheless, a better understanding of the factors that contribute to thermogenic adipocyte development and function is needed to fully harness the therapeutic potential of these cells.

Beige adipocytes are thermogenic adipocytes found in classically white fat depots and the developmental origins of these cells are still not fully understood (1). They have been shown to develop from progenitor cells distinct from those cells giving rise to white adipocytes (6), and cell surface markers such as CD81 are known to enrich beige progenitor cells from white adipose tissue via FACS (7). However, as evidence emerges showing that different programs can be activated to drive thermogenesis in adipocytes, it has become clear that there is heterogeneity among beige cells that may start at or before the preadipocyte stage (8). A better understanding of the signaling events underlying beige preadipocyte specification and/or proliferation may in turn be the key to understanding the factors that drive the development of beige adipocytes in response to environmental and metabolic challenges.

## Paracrine communication via IL-13 enhances beiging

In this issue of the *JCI*, Yesian and colleagues explored the signaling pathway mediated by the IL-13/IL-13 receptor α1 (IL-13/IL-13Rα1) axis. Notably, IL-13 signaling caused preadipocytes to more readily become metabolically active beige adipocytes (9). While the classical pathway to activate thermogenesis involves stimulation by 3 adrenergic receptors, which are activated by central sympathetic signaling in response to cold (10), thermogenic fat can also be activated by other means. In recent years, a number of studies have investigated the role that immune cells play in regulating brown and beige adipocytes (11). Macrophages have been found to be key players in regulating thermogenesis through both adrenergic and cholinergic signaling (12, 13); and recently, lymphocytes, including γ δ T cells and Tregs, have also emerged as important regulators of adipose tissue thermogenesis (14, 15).

Here, Yesian et al. have identified and clarified the role of type 2 innate lymphoid cell (ILC2) signaling in the regulation of beige preadipocytes and thus whole-body metabolism. The experiments specifically interrogated the role of type 2 cytokine IL-13 signaling through its receptor IL-13Rα1 in priming adipose progenitor cells to become beige adipocytes (9). While it has previously been suggested that the type 2 cytokine IL-4 also plays a role in beige fat biogenesis (16), Yesian and authors found that *Il4* whole-body–KO mice had no evident thermogenic defects, yet mice with whole-body *Il13* or *Il13ra1* KO showed clearly compromised activation of thermogenic responses in subcutaneous adipose tissue when challenged with cold or β3 adrenergic stimulation (9). The findings suggest that IL-13 may serve as the major type 2 cytokine in regulating beige fat differentiation.

**Figure 1:**
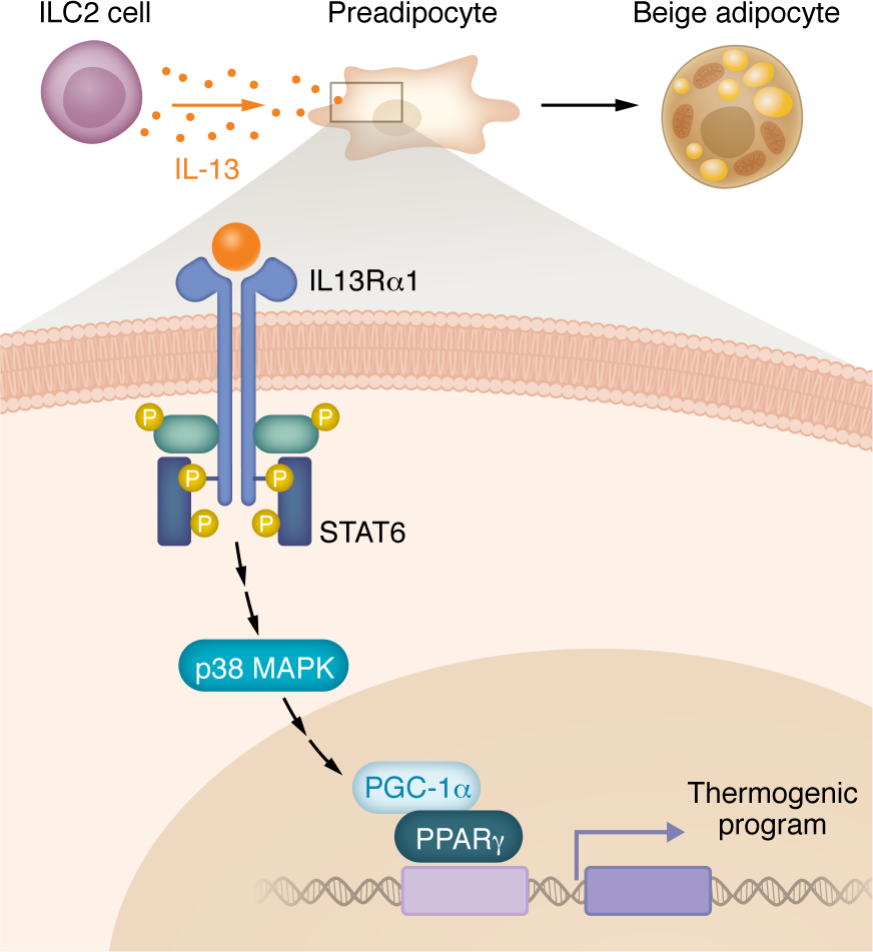
IL-13 signaling primes preadipocytes to differentiate into beige adipocytes. ILC2s secrete IL-13, which signals through IL-13Rα1 on preadipocytes. Subsequent activation of STAT6 and p38 MAPK recruits PGC-1α to PPARγ to drive a thermogenic transcriptional program. The expression of thermogenic genes promotes differentiation of the cells into beige adipocytes with elevated mitochondrial respiration capacity.

Investigating the mechanism by which IL-13 increases thermogenesis, Yesian and authors treated preadipocytes with IL-13. This treatment caused not only the differentiated adipocytes but also the undifferentiated preadipocytes to show increased mitochondrial respiration (9). In the preadipocytes, IL-13 signaled through STAT6 and p38 MAPK, which increased recruitment of PPARγ coactivator 1α (PGC-1α) to PPARγ and drove a thermogenic transcriptional program (9) (Figure 1). These data, therefore, convincingly demonstrate that IL-13 signaling drives preadipocytes toward a more thermogenic phenotype and that the adipocytes that differentiate from these preadipocytes are functional beige adipocytes, regulating whole-body energy homeostasis and metabolism.

It is striking that mature beige/brown adipocyte *Il13ra1*-KO (*bIl13ra1*-KO) mice displayed rather modest thermogenic defects, indicating that the beiging effect of IL-13 primarily takes place in preadipocytes. Furthermore, when *Prx1*^Cre^ was used to specifically knock out *Il13ra1* in subcutaneous preadipocytes, the preadipocyte *Il13ra1*-KO (*pIl13ra1*-KO) mice, when challenged with cold, showed reduced core body temperature and a blunted thermogenic response, even though the KO only affected subcutaneous adipose tissue where beige adipocytes were prominent, and not classical brown fat. This finding, along with the relative lack of a thermogenic defect in the *bIl13ra1*-KO mice, suggests that the effects mediated via IL-13/IL-13Rα1 in beige adipocytes are sufficient to increase thermogenesis and improve whole-body metabolism, despite the lack of involvement of classical brown fat (9). As studies continue to focus on the heterogeneity in beige adipocytes (17, 18), further work will need to reveal the role of different stimuli and progenitor subpopulations involved with increasing the content and activity of thermogenic beige adipocytes. It is of note that, in adult (20-week-old) *pIl13ra1*-KO mice, in which minimal thermogenic defects were detected, impaired glucose homeostasis was evident compared with the age-matched control mice. The observation highlights optimal beige fat function as an essential requirement for whole-body metabolic health and further suggests that much of the metabolic beneficial effects from beige adipocytes may be independent of their thermoregulatory function.

## Conclusion and future directions

Last, but not least, Yesian and authors showed multiple GWAS with hits for BMI and type 2 diabetes are found near the *IL13RA1* locus, suggesting that IL-13 signaling may play a role in weight and metabolic regulation in humans (9). The results echo a previous finding involving the fat mass and obesity–associated (*FTO*) gene variants that influence weight by decreasing thermogenesis in preadipocytes and mature beige adipocytes (19). While treatments that increase adipose thermogenesis have not had a strong effect on body weight thus far, these findings suggest that targeting preadipocyte and beige adipocyte thermogenesis can positively influence body weight in humans. The signaling pathway downstream of IL-13/IL-13Rα1 is mediated through PPARγ and high-affinity ligands for PPARγ, such as thiazolidinedione (TZD), have also been used clinically for treating type 2 diabetes (20). Future investigation will reveal whether further synergistic effects can be achieved when activating IL-13 and PPARγ together in the treatment of obesity and metabolic disorders. Furthermore, it has previously been shown that IL-13 signaling enhances exercise-elicited metabolic adaptation within skeletal muscle (21), suggesting that therapeutic approaches targeting IL-13 may hold great promise for improving systemic fitness through metabolic benefits in various tissues.

## Version 1 06/02/2025

Electronic publication
